# Glycine nutrition and biochemistry from an aquaculture perspective

**DOI:** 10.1093/af/vfae014

**Published:** 2024-09-05

**Authors:** Blaine A Suehs, Delbert M Gatlin, Guoyao Wu

**Affiliations:** Department of Ecology and Conservation Biology, Texas A&M University, College Station, TX 77843, USA; Department of Ecology and Conservation Biology, Texas A&M University, College Station, TX 77843, USA; Department of Animal Science, Texas A&M University, College Station, TX 77843, USA

**Keywords:** amino acids, glycine, fish, functional, metabolism, physiology, growth

**Implications**

- Glycine is a functional amino acid that serves in numerous essential physiologic and metabolic processes.
- Additional supplementation of glycine in the diet of aquatic species has been shown to impart beneficial effects.
- Future research should evaluate the biochemical mechanisms associated with glycine nutrition in fish.

## Introduction

### Current aquaculture practices and trends

Aquaculture represents the fastest-growing farmed food sector globally, surpassing total capture fisheries at approximately 90 million tons for human consumption (FAO, 2022). However, due to the rapid increase of farmed fish production to meet the protein demands of an increasing world population, marine feedstuffs (fishmeal and fish oil) are now considered finite resources and are transitioning to strategic ingredients in the diets of most carnivorous fish, and are largely omitted in the diets of omnivorous species such as Nile tilapia and channel catfish except in early life stages. This limited supply and increased demand has created a unique inequality in the market of these marine raw materials, as the 2023 price of fishmeal and fish oil exceeded USD 1,800/ton and USD 3,000/ton, respectively (FAO, 2022). As the price of marine protein and oils has increased dramatically, plant and animal byproducts have become foundational ingredients in aquatic diets for carnivorous and omnivorous fish species alike. Although plant-derived feedstuffs do have some negative characteristics, such as antinutritional factors and inadequate amino acid (AA) profiles, plant-protein feedstuffs (such as soybean meal, SBM) have become primary contributors to crude protein in many diets due to their relatively high protein content and marginal price. However, nutritionists must be cognizant of AA concentrations in diets with high inclusion levels of plant-protein feedstuffs to prevent AA deficiencies in fish.

### Amino acid properties and classification

Chemically, AAs are organic molecules that contain both amino and acid groups. Approximately 700 AAs have been identified to date; 20 are incorporated into protein in animals. While AAs serve as the building blocks of protein, they are also vital to many metabolic and physiological processes including cell signaling and nitric oxide (NO) production, lipid metabolism and bile salt production, protein (e.g., collagen) synthesis, and immunity (Wu 2022). Glycine (2-aminoacetic acid) is a prototype of an AA with enormous physiological functions. Notably, although intact protein is ingested in diets of farmed animals and fish, this macro-nutrient is inevitably broken down to AAs, di-, and tripeptides and absorbed through intestinal enterocytes by specific transmembrane transporters before being transported to extraintestinal tissues through portal circulation. Therefore, although overall protein intake often overshadows specific AA nutrition, AAs are vital to maintaining optimal health and survival of all living organisms.

Traditionally, AAs had been classified in animal nutrition based on growth performance and nitrogen balance with little regard for efficiencies in specific feed ingredient incorporation, age-specific differences in AA requirements of various species, or overall metabolic and physiological functionality. Specifically, nutritionally essential (indispensable) AAs are those AAs that cannot be synthesized or adequately synthesized de novo from their respective precursors and must be included in the diet to maintain homeostasis, health and, in growing animals, protein accretion in the body (Table 1). Alternatively, nutritionally nonessential (dispensable) AAs are those that can be synthesized in the body presumably in adequate amounts for growth and are not required in the diet (Table 1). While most fish nutrition disciplines utilize the traditional AA classification, novel and innovative strategies have been implemented to better characterize the need for AAs in the diets of farmed fish based on a wholistic approach to AA nutrition. Wu (2013) coined the “functional” AA philosophy, pioneering a new approach to AA supplementation in the diets of fish and other farmed animals based on overall benefits to metabolic and physiological processes and not exclusively growth. Under the functional AA concept, AAs classically deemed nonessential could impart beneficial effects when supplemented to diets (e.g., high-plant-protein diets). Thus, research on functional AAs [including those traditionally classified as nonessential (e.g., glycine)] is justified to enhance the field of aquaculture and fish nutrition.

**Table 1.** Traditional classification of nutritional essential (indispensable) or nonessential (dispensable) amino acids (AAs) as well as recently proposed functional amino acids in fish nutrition

Nutritionally essential AAs	Nutritionally nonessential AAs	Functional AAs
Arginine	Alanine	Arginine
Histidine	Asparagine	Aspartate
Isoleucine	Aspartate	Cysteine
Leucine	Cysteine*	Glutamate
Lysine	Glutamate*	Glutamine
Methionine	Glutamine*	Glycine
Phenylalanine	Glycine*	Leucine
Threonine	Hydroxyproline	Methionine
Valine	Proline*	Proline
	Serine	Taurine
	Taurine*	Tryptophan
	Tyrosine	Tyrosine

* Recognized as a species-dependent conditionally essential AA.

### Glycine properties and functionality

One such functional AA gaining recent research popularity is glycine (Wang et al., 2013). Glycine is the smallest and often perceived as the simplest AA but is the most abundant AA in animals including fish (Table 2). Interestingly, as in growing pigs (He et al. 2023a), glycine is among the most abundant AAs in the serum of juvenile hybrid striped bass (He et al. 2023b). In fish as in terrestrial animals, glycine can be formed from 4-hydroxyproline, serine, threonine, choline, and betaine primarily via multiple metabolic pathways (Wu, 2022). Biochemically, glycine is essential for syntheses of proteins (including collagen), glutathione (GSH), heme, glyco-bile conjugates, purines, and creatine (Table 3). To meet biological demands, glycine must be synthesized de novo from serine (via enzymatic serine hydroxymethyl transferase), choline (sarcosine formation), threonine (mitochondrial threonine dehydrogenase), and hydroxyproline (hydroxyproline-derived glyoxylate interconversion) even in fish (e.g., juvenile hybrid striped bass) fed a 60% fishmeal-based diet (Table 4). Although classified as nutritionally nonessential or conditionally essential, copious evidence of the vital functionality of glycine is seen throughout the literature, necessitating further research into this molecule.

**Table 2.** Composition of amino acids in the diet, plasma, and whole-body protein of hybrid striped bass

EAA	Diet^1^ (%)	Serum^2^ (µM)	Body protein^3^ (mg/g of BW)	NEAA	Diet^1^ (%)	Serum^2^ (µM)	Body protein^3^ (mg/g of BW)
Arginine	3.07	140 ± 8.6	9.80 ± 0.15	Alanine	2.96	554 ± 31	9.75 ± 0.11
Histidine	0.98	237 ± 15	3.49 ± 0.07	Asparagine	1.71	66 ± 2.7	5.14 ± 0.08
Isoleucine	1.80	148 ± 8.7	5.56 ± 0.05	Aspartate	2.51	28 ± 1.4	6.47 ± 0.11
Leucine	3.05	235 ± 12	9.76 ± 0.22	Cysteine	0.41	157 ± 9.3	2.01 ± 0.09
Lysine	3.20	176 ± 9.1	8.71 ± 0.15	Glutamate	3.90	67 ± 3.5	12.8 ± 0.29
Methionine	1.32	55 ± 2.8	4.01 ± 0.13	Glutamine	2.63	206 ± 10	8.11 ± 0.12
Phenylalanine	1.66	80 ± 2.2	5.71 ± 0.12	Glycine	2.95	292 ± 24	13.1 ± 0.33
Threonine	1.76	133 ± 6.0	5.73 ± 0.09	OH-proline	1.01	43 ± 2.0	3.03 ± 0.16
Tryptophan	0.46	29 ± 1.3	1.60 ± 0.06	Proline	2.46	231 ± 11	9.49 ± 0.22
Valine	2.09	264 ± 17	6.49 ± 0.17	Serine	1.86	172 ± 6.8	6.93 ± 0.13
Taurine	0.60	979 ± 54	—	Tyrosine	1.37	72 ± 2.9	4.12 ± 0.13

Values are mean ± SEM, *n* = 6. Adapted from Li et al. (2021) for 50-g hybrid striped bass fed a diet containing 60% fishmeal (on a dry-matter basis).

^1^The content of total AA in the diet.

^2^Blood samples were obtained at 24 h after the last feeding to obtain serum for amino acid analysis.

^3^Calculations were based on the molecular weights of amino acid residues (i.e., the molecular weight of an intact amino acid—18) in protein.

BW, body weight; EAA, nutritionally essential amino acids; NEAA, nutritionally nonessential amino acids; OH-Pro, 4-hydroxyproline (post-translational product of proline hydroxylation).

**Table 3.** Physiological roles of glycine in fishes (including hybrid striped bass)

Variable	Physiological roles of glycine and its metabolites
Direct action	Neurotransmitter; antioxidant; anti-inflammation; one-carbon metabolism
Precursor for syntheses	
Protein	Collagen (1/3 of the body proteins) and other tissue proteins
Serine	One-carbon metabolism; synthesis of phosphatidylserine and D-serine (neurotransmitter)
Porphyrins and heme	Hemoproteins (e.g., hemoglobin, myoglobin, catalase, and cytochrome *c*); production of carbon monoxide (a signaling molecule); storage of iron in the body
Bilirubin	Natural ligand of aryl hydrocarbon receptor in the cytoplasm
Creatine	Antioxidant, antiviral, energy metabolism, development of skeletal muscle and brain
Glutathione^2^	Free radical scavenger, antioxidant, immune response, regulation of gene expression
Nucleic acids	Coding for genetic information, gene expression, cell cycle and function,
Heme	An essential component of hemoglobin, myoglobin, and heme-containing enzymes

**Table 4.** Dietary provision of glycine and metabolic needs of juvenile hybrid striped bass (5.5 to 22.1 g of body weight) for glycine

Variable	Amounts of glycine (mg/fish over 4 weeks)
Glycine provision from a 60%-fishmeal diet [on a dry-matter (DM) basis]	491
Total glycine from diet containing 2.95% glycine (18.5 g feed DM/fish over 4 weeks)	546
Undigestible glycine (10%)	54.6
Glycine required for growth and metabolic function	834
Body weight gain (16.6 g/fish over 4 weeks; 13.1 mg glycine/g of body weight)	218
Glycine oxidation to carbon dioxide	447
Serine synthesis	62.5
Glycine utilization for creatine synthesis (1.57 mg creatine plus creatine-P in body)	31.0
Glycine utilization for purine synthesis	13.4
Glycine utilization for glutathione synthesis (0.492 mg/g of body weight)	8.2
Glycine utilization for heme synthesis	0.23
Loss to the surrounding water via the skin, gills, and urine	37.0
Glycine needed through endogenous synthesis from other amino acids and choline (i.e., 834 – 491)	343
Serine	13.2
Choline (via sarcosine)	5.1
Threonine (via threonine dehydrogenase)	6.4
4-Hydroxyproline [by subtraction; namely 343 – (13.2 + 5.1 + 6.4)]	318
[Dietary 4-hydroxyproline (considering 90% digestibility and that 85% of absorbed dietary 4-hydroxyproline is converted into glycine)]^1^	143

Growth data are taken from Jia (2019). Metabolic data were from our unpublished work. The hybrid striped bass (5.5 to 22.1 g) consumed 18.5 g of feed DM and gained 16.6 g of body weight during a 4-week period. ^1^ The diet contained 1.01% 4-hydroxyproline (on a DM basis). Glycine synthesis from endogenous protein (primarily collagen)-derived 4-hydroxyproline = 318 – 143 = 175 mg/fish over 4 weeks.

Glycine is recognized as an AA prevalent in fishmeal (approximately 6.58% dry weight) and animal protein feedstuffs such as poultry byproduct meal, but not so in SBM (2.72% dry weight) and other plant proteins (Li et. al., 2011). As fishmeal is increasingly replaced by plant protein to meet producer demands, glycine inclusion in practical diets could become deficient. While it is possible to completely replace fishmeal and fish oil in carnivorous fish diets, functional AAs must be supplemented when deficient in order to maintain the viability of the diet. Moreover, commercially relevant carnivorous fish species such as hybrid striped bass (*Morone chrysops* × *M. saxatilis*: HSB) and largemouth bass (*Micropterus salmoides*: LMB) are of particular interest for glycine supplementation when alternative protein feedstuffs are utilized (Rossi et al., 2021; He et al., 2023b; Li et al., 2023).

## Glycine Functions

### Protein and collagen synthesis

Glycine is the most abundant AA found in all collagen types (I–VII). Collagen accounts for approximately 30% of total proteins in the body. By weight (g/g), glycine, proline, and hydroxyproline combined totals 50% of collagen composition (Li and Wu, 2018). Moreover, collagen is the most abundant protein in most animal species comprising approximately 30% of total protein composition and plays key roles in hyperplasia, hypertrophy, skeleton structure, and tissue regeneration (Nimni and Harkness, 2018). Collagen fibrils are the primary molecules that provide support and rigidity to the extracellular matrices of connective tissues, such as bones, tendons, and skin. The closely packed spatial arrangement of specific collagen types offers optimal stability and protection of these tissues and is directly involved in tissue repair when injured (Ottani et al., 2001). Interestingly, fish waste (bones, skin, and scales) is a byproduct that is inevitably high in collagen protein. Recent advancements in biomedical sciences have begun to utilize fish-derived collagen to mediate physiological responses, such as anti-inflammatory modulation of macrophages through the activation of glycine receptors, as well as tissue repair enhancement through the localization of collagen in wounds (Subhan et al., 2021). Therefore, not only is collagen essential for the fish to increase connective tissue mass in a production setting but it also has downstream applications for the enhancement of animal health. This represents a shift in the industry preferred origin of collagen from either bovine or porcine sources. For example, channel catfish skin-derived collagen offers a similar AA profile, with glycine residues being the most abundant at approximately 25%, to the porcine collagen source, while still maintaining beneficial physiological properties and functions of the mammalian counterpart (Liu et al., 2007).

### Glutathione synthesis

Reduced glutathione (GSH) is the most abundant low-molecular-weight antioxidant in organisms serving key functions in oxidative stress mitigation through glutathione-*S*-transferases (GST) and glutathione peroxidases (GPx) (Gul et al., 2000). GSH is synthesized from glycine, cysteine, and glutamate through the γ-glutamyl cycle via the enzymes γ-glutamyl cysteine synthetase and glutathione synthetase (Gul et al., 2000). GSH (the reduced form) is converted to oxidized GSH (GSSG) when GSH is oxidized by reactive oxygen species (ROS) and peroxides (including peroxides of polyunsaturated fatty acids). NADPH is then necessary to reduce GSSG back to GSH, providing cooperativity between GSH and the pentose phosphate pathway (Wu, 2022). Therefore, GSH is heavily involved in the prevention of oxidative stress from oxidative molecules, transportation, hypoxia, and hyperammonemia. Most recently, He et al. (2024) reported that increasing the content of glycine from 2.2% to 3.2% and 4.2% (on a DM basis) increased GSH synthesis and availability in tissues of phase I (5-40 g) and phase II (110-240 g) hybrid striped bass.

In teleost fish, GSH is present in most tissues, with heavy localization in the liver allowing adequate protection against electrophilic molecules (Li et al., 2021). Among all tissues of hybrid striped bass examined, the liver is most active in GSH synthesis, followed by the intestine (He et al., 2024). Additionally, glycine supplementation in the diet of common carp (*Cyprinus carpio*), Nile tilapia, and Beluga sturgeon (*Huso huso*) elevated the circulating levels of GSH during times of transportation oxidative stress, while simultaneously lowering plasma cortisol levels (Xie et al., 2016; Hoseini et al., 2022a, 2022b). Similarly, glycine supplementation reduced hyperammonemia in common carp by reducing overall toxicity as well as enhancing oxidative responses (Hoseini et al., 2022b). Glycine supplementation provides a less expensive and better protected alternative to GSH supplementation but imparts similar overall effects.

### One carbon unit metabolism and purine synthesis

Cytoplasmic folate-mediated one carbon unit metabolism is an interconnected metabolic network that is responsible for purine and thymidylate synthesis, and the methylation of homocysteine (a biologically toxic molecule) to methionine (Fox and Stover, 2008). In this process, mitochondrial glycine is synthesized from serine, with tetrahydrofolate (THF) being converted into 5,10-methylene-THF. The latter is metabolized to 10-formyl-THF and then formate, which is transported to the cytosol for purine synthesis (Fox and Stover, 2008). In addition, glycine is interconverted into serine through the action of serine hydroxymethyl transferase, thereby participating in one carbon unit metabolism that is essential for nucleic acid synthesis, cell signaling, and gene expression. Unfortunately, the specific mechanisms of these processes are understudied in fish.

### Creatine, heme, and bile acid synthesis

Phosphorylated creatine is a molecule responsible for maintaining physiological ATP balance in cells during increased energetic demands such as the swimming of fish. While mammals synthesize creatine from glycine, l-arginine, and l-methionine in the liver, kidney, and pancreas, skeletal muscle is the major organ for creatine synthesis in teleost fish such as hybrid striped bass (He et al., 2024). Numerous studies have evaluated effects of dietary creatine supplementation in aquatic species, namely, hybrid striped bass (Janes et al., 2023), red drum (Burns and Gatlin III, 2019), and rainbow trout (McFarlane et al., 2001). Because creatine is absent from plant-sourced ingredients but is present in fishmeal and other animal-sourced products (Li et al., 2021), the inclusion of creatine in plant-based diets of fish may spare glycine for protein and GSH synthesis as well as lean tissue growth, and vice versa (He et al., 2024).

Glycine along with succinyl-CoA initiates heme biosynthesis through the actions of pyridoxal phosphate-dependent δ-aminolevulinate synthase (Wu, 2022). Ultimately, heme is vital to the regulation of hemoglobin and myoglobin formation essential for oxygen transport in the blood and oxygen storage in skeletal and cardiac muscles (Ueki and Ochiai, 2006). Comparatively, little work has been published on structural differences of fish heme compared to that of mammalian heme; however, Ueki and Ochiai (2006) reported the absence of the D helix in tuna myoglobin that could have downstream consequences on chemical and physical properties of the molecule as compared to mammals.

Bile acid conjugation with glycine or taurine is necessary for the efficient digestion and absorption of dietary lipids in the gastrointestinal tract of animals including fish (Li et al., 2021). As seen in mammals, supplemented glycine has been demonstrated to increase overall lipid retention in HSB and LMB (Rossi Jr et al., 2021; He et al., 2023b; Li et al., 2023). Thus, glycine is essential in regulating lipid metabolism along with intracellular protein turnover. Several reports indicate a lower affinity of certain carnivorous fish species for the utilization of glycine-conjugated bile acid, with taurine serving as the dominant conjugator (Romano et al., 2020). Although understudied, species-specific differences in bile acid conjugation appear to exist particularly between marine and freshwater fish.

### Overall growth and health

**Figure 1.**
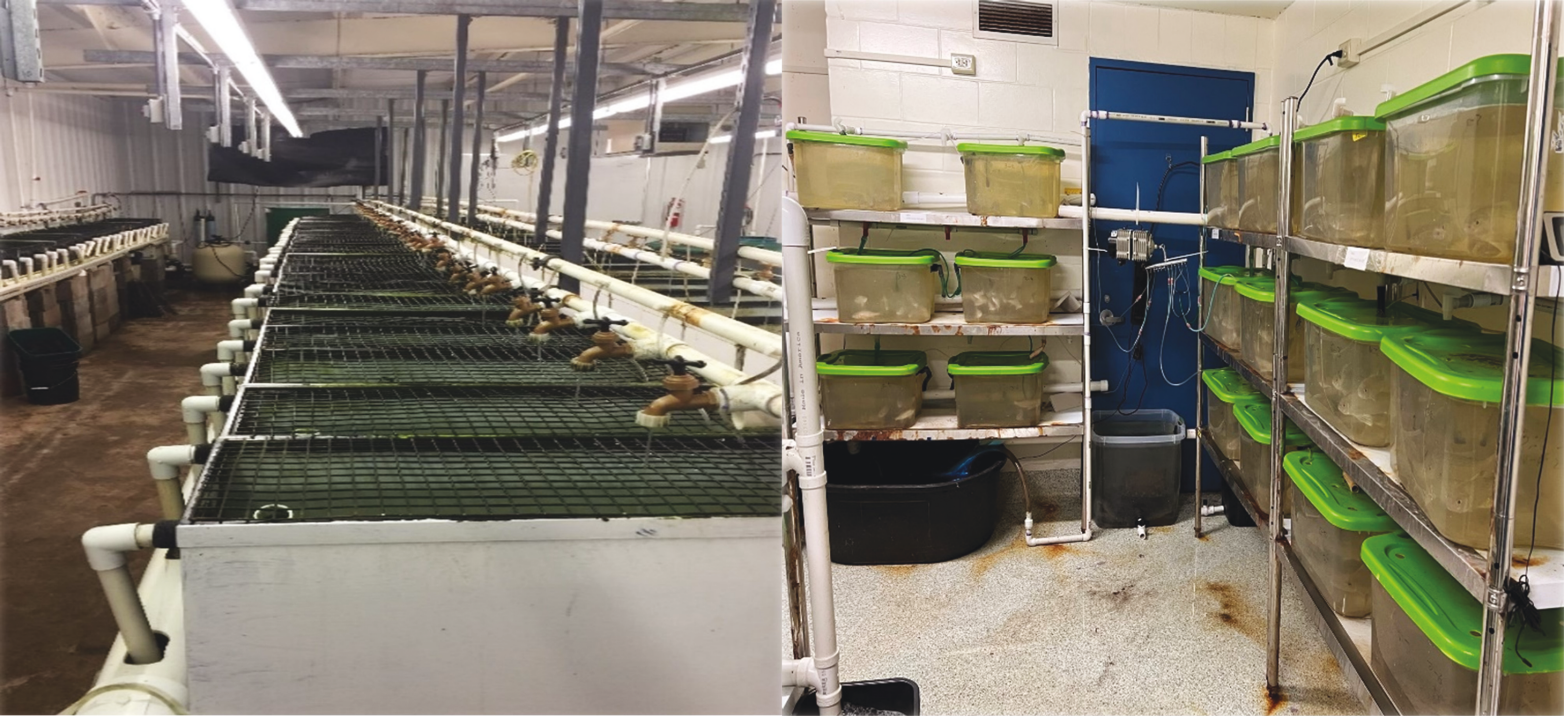
Fish housing and experimental systems located at the Aquacultural Research and Teaching Facility (Left) and Amino Acids Laboratory (Right) located at Texas A&M University.

Numerous published studies have evaluated the efficacy of glycine supplementation in aquatic diets for omnivorous and carnivorous fish species as fishmeal is replaced with plant-protein ingredients (Figure 1). Of note, carnivorous species such as HSB and LMB have been reported to have increased growth response when supplemented with glycine in high-plant-protein diets (Rossi et al., 2021; He et al., 2023b; Li et al., 2023), as reported for pigs (He et al. 2023a). Additionally, intestinal health was also improved when glycine was supplemented at 1% or 2% of diet in each of these trials. For example, glycine-supplemented HSB exhibited increases in nutrient absorption and the efficiency of dietary AAs for protein deposition (nitrogen retention) (Li et al., 2023). Similarly, rainbow trout, a cold-water carnivorous fish, also exhibited increased growth percentage and muscle accretion when supplemented with glycine at 2% of the diet (Belghit et al., 2023). Historically, glycine supplementation has been seen as a method of palatability enhancement for soybean-meal-based diets in carnivorous species (Li et al., 2021); however, recent advances in glycine metabolism and nutrition potentially illustrate a more functional mechanism responsible for its action than originally believed (Li et al., 2023; He et al., 2024).

Similarly, evidence has been reported on beneficial effects of glycine supplementation to omnivorous fish species as well. Research on Nile tilapia (Xie et al., 2016), common carp, and beluga sturgeon (Hoseini, et al., 2022a) has all revealed significantly increased production performance when fed glycine-supplemented diets. Additionally, immunomodulation, namely an increase in innate immune superoxide dismutase, has also been demonstrated in multiple species of fish (Rossi Jr et al., 2021; Belghit et al., 2023). This conclusion follows closely with the function of glycine in glutathione production and antioxidative defense (Wu, 2022).

Due to traditional classifications of glycine as a nutritionally nonessential AA, its dietary requirement for aquatic species is lacking. However, recent publications suggest a requirement of dietary glycine to be 3.0% to 4.0% of DM, depending on the feeding behavior of the species (Table 5). Due to the low content of glycine in plant proteins, practical diets (containing approximately2.0-2.3% glycine; He et al., 2023; Li et al., 2023) often lack the required amount of glycine for optimal growth and physiological processes (including ammonia detoxification; Hoseini et al., 2024) of both carnivorous and omnivorous fish species. Thus, there is a sufficient body of evidence that supports the supplementation of glycine as a functional AA in the diets of agriculturally important fish species.

**Table 5.** Experimental results of published studies regarding glycine supplementation in the diets of carnivorous and omnivorous fish species

Species	Feeding behavior	Supplementation level (% dry weight)	Experimental dietary glycine (% dry weight)	Type of study	Results (compared to control diet)	References
Beluga Sturgeon (*Huso huso*)	Carnivorous	0.25-1.0%	2.0-2.8%	Growth/immunological	Increased: • Innate immune response • GSH synthesis Decreased: • Plasma cortisol	Hoseini et al. (2022a)
Common Carp (*Cyprinus carpio)*	Omnivorous	0.5-1.0%	2.5-3.0%	Metabolic	Suppression of: • Hyperammonemia • Stress response • Oxidative stress	Hoseini et al 2022b, 2024)
Common Carp (*Cyprinus carpio*)	Omnivorous	0.5-1.0%	2.1-2.8%	Growth/ Immunological	Increased: • Weight gain • GSH Synthesis • Immune response • Blood neutrophils and monocytes	Abbasi et al. (2023)
Hybrid Striped Bass (*Morone chrysops × M. saxatilis)*	Carnivorous	1.0-2.0%	3.1-4.1%	Growth	Increased: • Weight gain • Intestinal health • Nutrient retention	Li et al. (2023) and He et al., (2023b)
Hybrid Striped Bass (*Morone chrysops × M. saxatilis)*	Carnivorous	1.0-2.0%	3.2-4.2%	Biochemical	Increased Synthesis of: • Creatine • GSH	He et al. (2024)
Largemouth Bass (*Micropterus salmoides*)	Carnivorous	2.0%	3.74%	Growth	Increased: • Weight gain • Protein and AA retentions • Innate immune response	Rossi Jr. et al. (2021)
Nile Tilapia (*Oreochromis niloticus*)	Omnivorous	0.5%	1.94%	Growth/ Immunological	Increased: • Weight gain • Anti-oxidative capacity	Xie et al. (2016)
Rainbow Trout (*Oncorhynchus mykiss*)	Carnivorous	1.0%	2.7%	Growth	Increased: • AA apparent digestibility • Lipid apparent digestibility	Belghit et al. (2023)

## Conclusion and perspectives

Glycine is the most abundant AA in animals including fish and plays important roles in their nutrition, growth, immune response, osmoregulation, and antioxidative defense. As a vital functional AA, glycine is required for the synthesis of molecules with enormous physiological significance, such as protein, heme, creatine, glutathione, bile salt, purines, and participates in one-carbon unit metabolism and ammonia removal. To meet the high requirements of the fish (even those fed a conventional 60% fishmeal-based diet) for glycine, it must be synthesized in the body at the rate of 41% of total glycine requirement. When fed plant-based diets that generally contain lower amounts of glycine, its endogenous synthesis is insufficient to meet the metabolic needs of fish for maximal growth or optimal health. Emerging evidence shows that glycine is a nutritionally essential AA for growing fish as for pigs. Although much research has been done on practical applications of glycine supplementation particularly in plant-protein diets, little has been documented on the molecular or cellular actions of glycine as well as glycine synthesis in fish when compared to terrestrial mammals. Thus, basic biochemical research is warranted to further the understanding of fish nutrition and comparative biochemistry. Namely, information on the intestinal transport of glycine, enterocyte and extraintestinal utilization of glycine, and the regulation of nutrient metabolism by glycine is lacking in the current body of literature. Additionally, glycine has been demonstrated to increase body weight and overall skeletal muscle mass in fish, but studies regarding the regulation of intracellular protein turnover (e.g., MTOR/rapamycin signaling) are grossly underrepresented in aquaculture. Increased basic knowledge of glycine nutrition in fish, with respect to species-specific variations, will aid in not only better understanding fish nutrition but also developing new and effective nutritional strategies (e.g., dietary glycine supplementation) to improve the growth performance (including feed efficiency) of fish and aid in sustaining global aquaculture.

## About the Authors

 **Blaine A. Suehs** is a PhD student in Dr. Delbert Gatlin’s Fish Nutrition Laboratory and Dr. Guoyao Wu’s Amino Acids Laboratory at Texas A&M University. He received his undergraduate degree in Wildlife and Fisheries Sciences as well as his masters degree in Aquaculture and Fish Nutrition from Texas A&M University. Throughout his graduate term, he has been employed through Texas A&M Agrilife Research on a research assistant fellowship in addition to several teaching assitantship roles. His research interests include areas related to warm water fish nutrition and health, specifically as it relates to fish meal substitution, alternative protein ingredients, and amino acid nutrition.

 **Dr. Guoyao Wu** is a University Distinguished Professor of Animal Science at Texas A&M University. His research focuses on the biochemistry, nutrition, and physiology of amino acids and related nutrients in animals (including pigs, sheep, cattle, rats, chickens, and fish) at genetic, molecular, cellular, and whole-body levels. He has published 768 papers in peer-reviewed journals, 99 chapters in books, and two text/reference books [*Principles of Animal Nutrition* (2018, CRC Press) and *Amino Acids: Biochemistry and Nutrition, 2*^*nd*^*edition* (2022, CRC Press)]. He has edited seven books on nutrition and metabolism for Springer and Elsevier. He has also served as an editor for *Amino Acids*, *Frontiers of Bioscience*, *Journal of Nutritional Biochemistry*, and *Encyclopedia of Animal Nutrition.* He is an elected fellow of the American Association for the Advancement of Science.

 **Delbert M. Gatlin III** is a Regents Professor in the Department of Ecology and Conservation Biology and member of the Intercollegiate Faculty of Nutrition at Texas A&M University. He has had an academic appointment with Texas A&M University since 1987. Dr. Gatlin earned a BS in fisheries/aquaculture from Texas A&M University in 1980 and a PhD in nutritional biochemistry from Mississippi State University in 1983. His research program encompasses many different aspects of nutrition including determination of requirements for and metabolism of various nutrients, as well as development and evaluation of diet additives, formulations and feedstuffs for various fish species including channel catfish, hybrid striped bass, red drum, and tilapia. He was a Nutrition Section Editor for the journal *Aquaculture* from 2009 through 2023 and the Editor-In-Chief from 2019 through 2023. He also was vice chair of the Committee on Nutrient Requirements of Fish and Shrimp of the National Academies from 2009 to 2010. He has co-authored 325 peer-reviewed journal articles, 19 book chapters and 5 books.

**Corresponding authors**: bsuehs19@tamu.edu, d-gatlin@tamu.edu, or g-wu@tamu.edu
